# HIV treatment failure in tertiary care: clinical epidemiology and preliminary mechanistic characterisation

**DOI:** 10.3389/fpubh.2026.1777588

**Published:** 2026-04-01

**Authors:** Peter Mac Asaga, Axel Kroeger, Sunday Omilabu, Philomena E. Airiohuodion, Arthur Oragwa, Deborah Buba, Chukwuma Anyaike

**Affiliations:** 1 Institute for Infection Prevention and Control, Medical Center, Faculty of Medicine, University of Freiburg, Freiburg, Germany; 2 Centre for Medicine and Society, Universitatklinikum Freiburg, Freiburg, Germany; 3 College of Medicine, University of Lagos, Lagos, Nigeria; 4 Faculty of Veterinary Medicine, University of Jos, Jos, Nigeria; 5 Federal Ministry of Health, Abuja, Nigeria

**Keywords:** drug resistance, HIV, immune activation, Nigeria, sub-Saharan Africa, treatment failure, viral suppression

## Abstract

**Background:**

Despite antiretroviral therapy (ART) scale-up in sub-Saharan Africa, treatment failure remains a significant challenge. We characterised virological and immunological outcomes among people living with HIV (PLHIV) attending tertiary care facilities in Nigeria, with exploratory analysis of potential mechanistic factors.

**Methods:**

This multi-centre cross-sectional study enrolled 517 HIV-positive adults from four Nigerian tertiary facilities between January 2019 and December 2021. Primary outcomes included viral load suppression (<1,000 copies/mL) and CD4 count. Exploratory mechanistic analyses examined drug resistance mutations (*n* = 50), immune activation markers (*n* = 40), and inflammatory biomarkers (*n* = 35) in pilot subsets.

**Results:**

Among 412 participants with viral load data, only 111 (26.9%; 95% CI 22.7–31.5) achieved viral suppression, substantially below the UNAIDS 95% target. Of 387 with CD4 data, 149 (38.5%; 95% CI 33.6–43.6) had severe immunodeficiency (<200 cells/μL). Among 346 participants with complete data, discordant responses were common: 25.7% showed virological failure with preserved immunity, while 6.6% had immunological failure despite viral suppression. In pilot mechanistic subsets, 86% of viraemic participants harboured drug resistance mutations, with M184V (62%) and K103N (54%) predominating. CD8 T-cell activation (CD38+HLA-DR+) was significantly elevated in viraemic versus suppressed participants (median 28.6% vs. 12.4%; *p* < 0.001), correlating inversely with CD4 count (*ρ* = −0.46; *p* < 0.01).

**Conclusion:**

HIV treatment outcomes at Nigerian tertiary facilities fall substantially short of global targets. The high prevalence of discordant immune-virological responses and preliminary evidence of drug resistance and immune activation suggest multiple interacting pathways to treatment failure. Larger mechanistic studies are warranted to inform targeted interventions.

## Introduction

The global HIV response has achieved remarkable progress, with 39.9 million people living with HIV (PLHIV) worldwide and 30.7 million receiving antiretroviral therapy (ART) by 2023 (1, 2). Sub-Saharan Africa, home to approximately 26 million PLHIV, has made substantial strides toward the UNAIDS 95-95-95 targets, with several countries including Botswana, Eswatini, and Rwanda achieving epidemic control (3, 4). However, significant gaps persist, particularly regarding the third 95—viral load suppression among those on treatment.

Nigeria, with an estimated 1.9 million PLHIV, represents the second-largest HIV burden globally (5). Recent programmatic data suggest improving cascade performance, with national estimates approaching 87-98-95 toward the 95-95-95 targets (6). However, these aggregate figures may mask substantial heterogeneity across facilities and populations. Tertiary care facilities, which often receive complex cases and patients failing first-line therapy, may experience different treatment outcomes than primary care settings.

Treatment failure in HIV is multifactorial, potentially involving drug resistance, suboptimal adherence, pharmacokinetic factors, and host immune responses (7, 8). Chronic immune activation and inflammation persist even during suppressive ART and are associated with adverse clinical outcomes (9–14). Understanding the relative contributions of these factors in specific clinical contexts is essential for developing targeted interventions.

We conducted a multi-centre study to characterise virological and immunological outcomes among PLHIV attending Nigerian tertiary care facilities. Primary objectives were to determine rates of viral suppression and immunodeficiency, and to characterise discordant immune-virological responses. Secondary exploratory objectives examined potential mechanistic contributors to treatment failure, including drug resistance mutations, immune activation, and inflammatory biomarkers in pilot subsets.

## Methods

### Study design and setting

This multi-centre cross-sectional study was conducted at four Nigerian tertiary healthcare facilities: Abia State University Teaching Hospital (ABSUTH) Aba (Southern Nigeria), Federal Medical Centre (FMC) Makurdi and FMC Keffi (Central Nigeria), and Baru Diko Teaching Hospital Kaduna (Northern Nigeria). Participants were enrolled between January 2022 and December 2023. The study was approved by the institutional review boards of all participating facilities, and written informed consent was obtained from all participants.

### Participants

Adults (≥18 years) attending HIV care services at participating facilities were eligible for enrolment. HIV status was confirmed using the national HIV testing algorithm. Participants with confirmed HIV infection were included regardless of ART status or duration. Exclusion criteria included inability to provide informed consent and acute intercurrent illness at the time of enrolment.

### Clinical and laboratory assessments

Demographic and clinical data were collected using standardised case report forms. Plasma HIV-1 RNA viral load was quantified using the Roche COBAS AmpliPrep/COBAS TaqMan HIV-1 Test (lower limit of detection 40 copies/mL). CD4+ T-lymphocyte counts were determined by flow cytometry (BD FACSCount or FACSCalibur). Viral suppression was defined as <1,000 copies/mL per WHO guidelines. Severe immunodeficiency was defined as CD4 <200 cells/μL.

### Exploratory mechanistic analyses (pilot subsets)

Drug resistance genotyping was performed in a convenience subset of 50 viraemic participants using population-based Sanger sequencing of the HIV-1 pol gene. Sequences were analysed using the Stanford HIV Drug Resistance Database. Immune activation was assessed in 40 participants by flow cytometry, measuring CD38 and HLA-DR co-expression on CD4+ and CD8+ T cells. Inflammatory biomarkers (IL-6, TNF-α, CRP) were quantified by ELISA in 35 participants. These analyses were exploratory and hypothesis-generating given the small sample sizes.

### Statistical analysis

Categorical variables were summarised as frequencies and percentages with 95% confidence intervals calculated using the Wilson method. Continuous variables were expressed as medians with interquartile ranges (IQR). Between-group comparisons used Mann–Whitney *U* tests for continuous variables and chi-square or Fisher’s exact tests for categorical variables. Correlations were assessed using Spearman’s rank coefficient. Multivariable logistic regression examined factors associated with viral non-suppression, adjusting for age, sex, duration on ART, pregnancy status, and region, with site included as a fixed effect. Analyses were performed using Stata 17.0 (StataCorp, College Station, TX). A two-sided *p* < 0.05 was considered statistically significant.

## Results

### Study population

**Figure 1:**
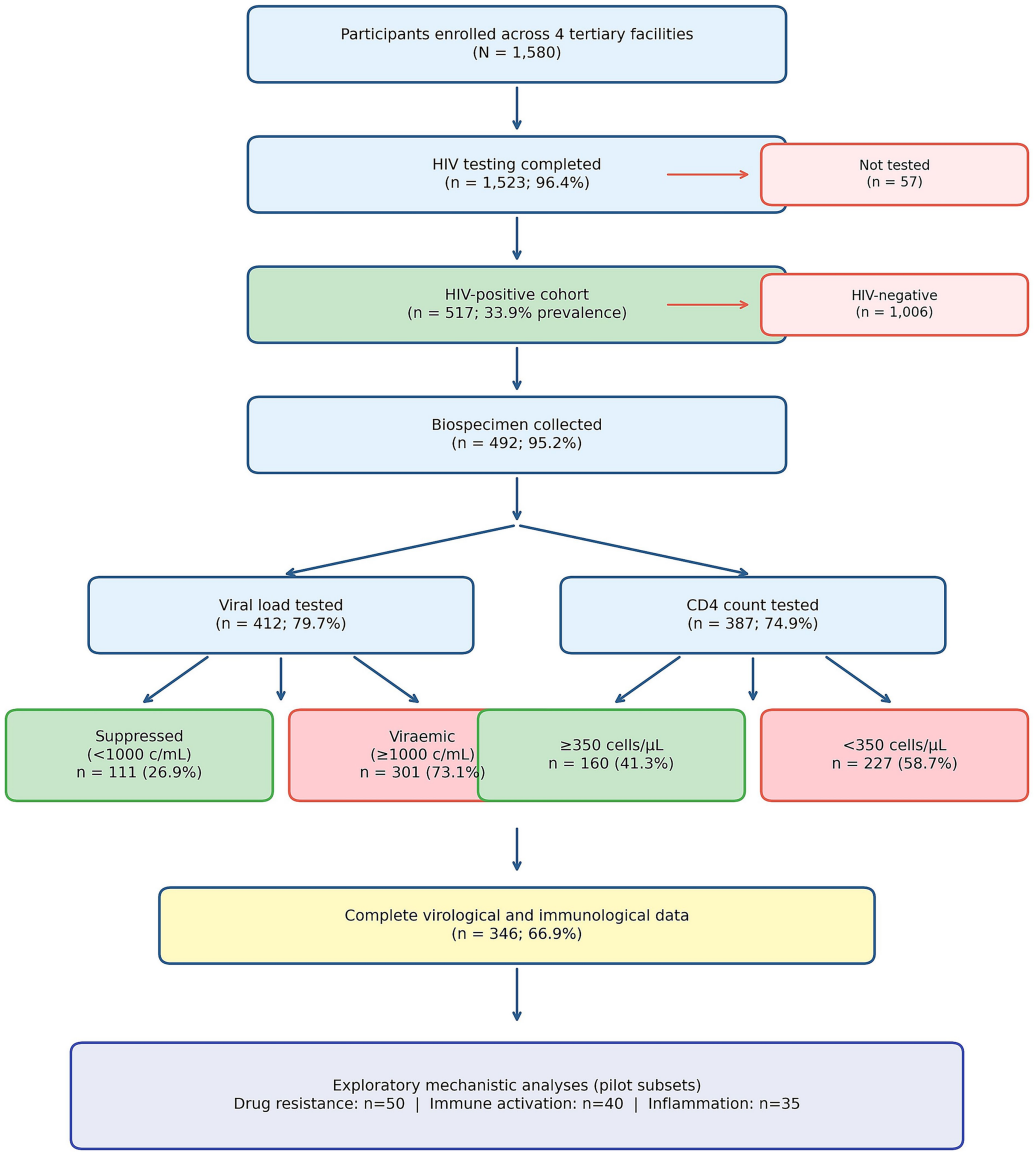
Study flow diagram. STROBE-compliant flow diagram depicting participant recruitment and data availability across four Nigerian tertiary care facilities. Of 1,580 participants enrolled, 1,523 (96.4%) completed HIV testing, yielding 517 HIV-positive individuals (33.9% prevalence). Viral load data were available for 412 participants (79.7%) and CD4 count data for 387 participants (74.9%). Complete immunovirological data enabling treatment response classification were available for 346 participants (66.9%). Exploratory mechanistic analyses were performed in pilot subsets: drug resistance genotyping (*n* = 50), immune activation (*n* = 40), and inflammatory biomarkers (*n* = 35).

Of 1,580 participants enrolled, 1,523 (96.4%) completed HIV testing, yielding 517 HIV-positive individuals (33.9% prevalence). The cohort was predominantly female (362/517; 70.0%), with a median age of 38 years (IQR 31–46). Among women, 86 (23.8%) were pregnant. Participants were distributed across Northern (242; 46.8%), Central (207; 40.0%), and Southern (68; 13.2%) Nigeria. Biospecimen collection was successful in 492 participants (95.2%). Viral load data were available for 412 participants (79.7%), CD4 data for 387 (74.9%), and complete immunovirological data for 346 (66.9%) (Table 1; Figure 1).

**Table 1:** Study population characteristics (*N* = 517).

Characteristic	*n*	%	95% CI
Sex			
Female	362	70.0	65.9–73.9
Male	155	30.0	26.1–34.1
Age, years (median, IQR)	38	—	31–46
Pregnancy status (among women)			
Pregnant	86	23.8	19.5–28.5
Non-pregnant	276	76.2	71.5–80.5
Region			
Southern Nigeria	68	13.2	10.4–16.4
Central Nigeria	207	40.0	35.8–44.4
Northern Nigeria	242	46.8	42.5–51.2
Testing completeness			
Viral load available	412	79.7	75.9–83.1
CD4 count available	387	74.9	70.9–78.5
Both VL and CD4 available	346	66.9	62.7–70.9

IQR, interquartile range; VL, viral load.

### Virological outcomes

Among 412 participants with viral load data, only 111 (26.9%; 95% CI 22.7–31.5) achieved viral suppression (<1,000 copies/mL), with 43 (10.4%) having undetectable viral load (<40 copies/mL). The remaining 301 participants (73.1%; 95% CI 68.5–77.3) had persistent viraemia, including 127 (30.8%) with very high viral loads (≥100,000 copies/mL). Median viral load among non-suppressed participants was 45,230 copies/mL (IQR 8,450–185,000) (Table 2). These suppression rates fall substantially below the UNAIDS target of 95% and recent Nigerian programmatic estimates (6).

**Table 2:** Virological and immunological outcomes.

Outcome	*n*	%	95% CI
Virological outcomes (*n* = 412)			
Viral suppression (<1,000 copies/mL)	111	26.9	22.7–31.5
Undetectable (<40 copies/mL)	43	10.4	7.7–13.9
Persistent viraemia (≥1,000 copies/mL)	301	73.1	68.5–77.3
High-level (≥100,000 copies/mL)	127	30.8	26.4–35.6
Median VL, non-suppressed (copies/mL)	45,230	IQR:	8,450–185,000
Immunological outcomes (*n* = 387)			
CD4 ≥500 cells/μL	90	23.3	19.1–28.0
CD4 350–499 cells/μL	70	18.1	14.4–22.3
CD4 200–349 cells/μL	78	20.2	16.3–24.6
CD4 < 200 cells/μL (severe immunodeficiency)	149	38.5	33.6–43.6
Median CD4 count (cells/μL)	285	IQR:	145–456

VL, viral load; IQR, interquartile range.

### Immunological outcomes

Among 387 participants with CD4 data, 149 (38.5%; 95% CI 33.6–43.6) had severe immunodeficiency (CD4 <200 cells/μL), including 53 (13.7%) with profound immunodeficiency (<100 cells/μL). Only 90 participants (23.3%) had CD4 counts ≥500 cells/μL indicating good immune function. Median CD4 count was 285 cells/μL (IQR 145–456). These findings indicate a high burden of advanced HIV disease in this population, consistent with recent estimates suggesting 1.8–1.9 million people in sub-Saharan Africa live with advanced HIV disease (4, 7).

### Discordant immune-virological responses

**Figure 2:**
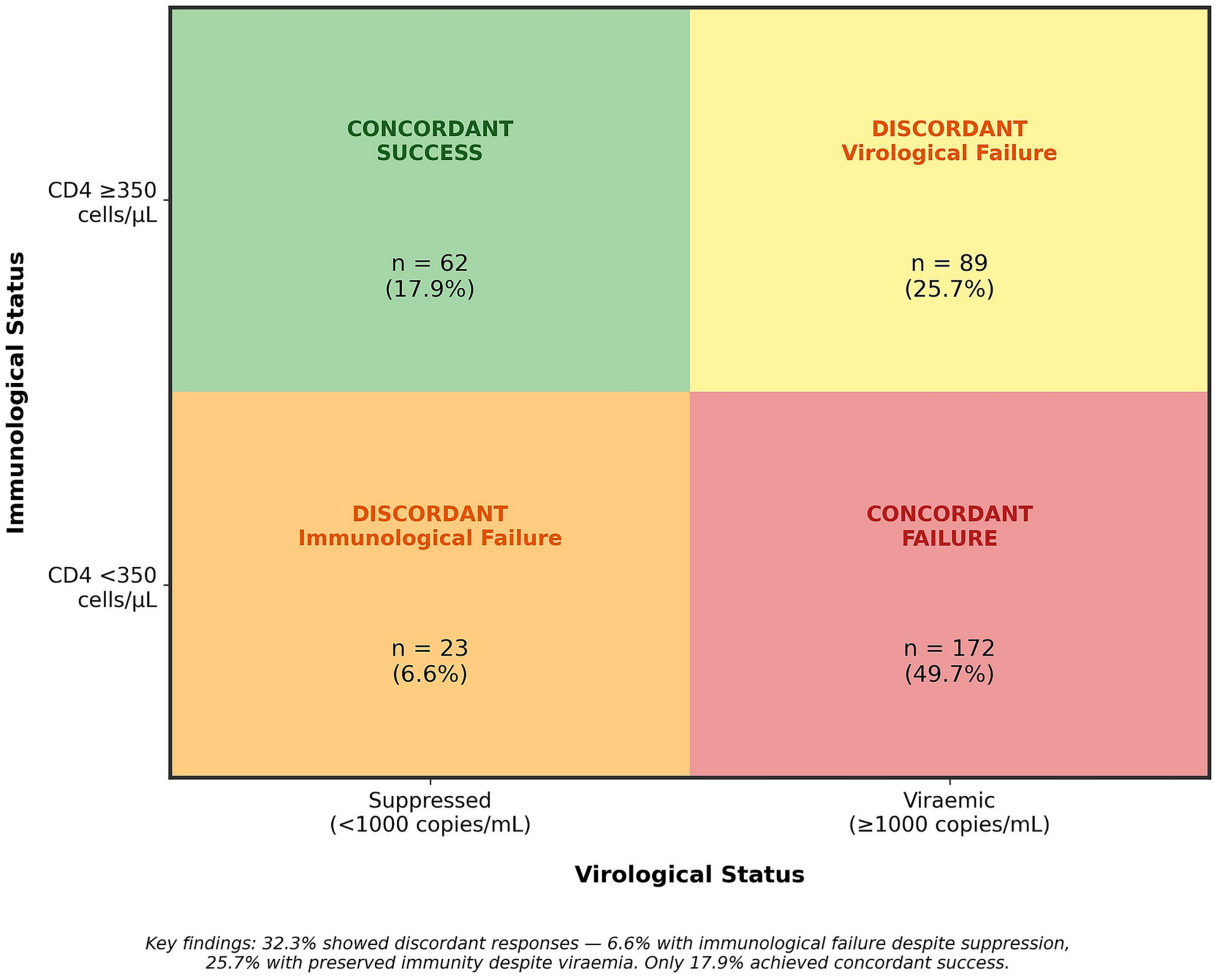
Treatment response patterns. Matrix representation of concordant and discordant immune-virological responses among 346 participants with complete data. Concordant success: viral suppression with CD4 ≥350 cells/μL. Concordant failure: viraemia with CD4 <350 cells/μL. Discordant immunological failure: viral suppression with incomplete immune recovery. Discordant virological failure: viraemia with preserved CD4 counts. Notably, 32.3% of participants exhibited discordant responses.

Among 346 participants with complete viral load and CD4 data, treatment response patterns revealed substantial discordance (Table 3). Only 62 participants (17.9%; 95% CI 14.0–22.4) achieved concordant success (viral suppression with CD4 ≥350 cells/μL), while 172 (49.7%; 95% CI 44.3–55.1) had concordant failure. Notably, 89 participants (25.7%; 95% CI 21.2–30.7) demonstrated discordant virological failure—persistent viraemia despite preserved CD4 counts (≥350 cells/μL). Conversely, 23 participants (6.6%; 95% CI 4.3–9.9) showed discordant immunological failure—inadequate CD4 recovery despite viral suppression. Among virologically suppressed participants (*n* = 85), 27.1% had incomplete immune recovery. Among viraemic participants (*n* = 261), 34.1% maintained preserved immunity (Figure 2).

**Table 3:** Treatment response patterns (*n* = 346 with complete data).

Response category	Virological status	Immunological status	*n* (%)	95% CI
Concordant success	Suppressed	CD4 ≥350 cells/μL	62 (17.9)	14.0–22.4
Concordant failure	Viraemic	CD4 <350 cells/μL	172 (49.7)	44.3–55.1
Discordant immunological failure	Suppressed	CD4 <350 cells/μL	23 (6.6)	4.3–9.9
Discordant virological failure	Viraemic	CD4 ≥350 cells/μL	89 (25.7)	21.2–30.7

Among virologically suppressed (*n* = 85): 27.1% had incomplete immune recovery. Among viraemic (*n* = 261): 34.1% maintained preserved immunity.

### Exploratory mechanistic findings

*Note: The following analyses were performed in small pilot subsets and should be interpreted as hypothesis-generating.*

Drug resistance genotyping in 50 viraemic participants revealed that 43 (86%) harboured at least one resistance-associated mutation. NRTI mutations were common, with M184V/I detected in 31 (62%) and K65R in 14 (28%). NNRTI mutations included K103N in 27 (54%) and Y181C in 11 (22%). Multi-class resistance affecting ≥2 drug classes was present in 19 participants (38%). These mutation patterns are consistent with regional surveillance data from East and Southern Africa (15–18).

Immune activation analysis in 40 participants showed significantly elevated CD8+ T-cell activation (CD38+HLA-DR+ co-expression) in viraemic compared to suppressed participants (median 28.6% vs. 12.4%; *p* < 0.001). CD8 activation correlated inversely with CD4 count (Spearman *ρ* = −0.46; *p* < 0.01), consistent with the established role of chronic immune activation in CD4 depletion (9, 13, 14, 19).

Inflammatory biomarker analysis in 35 participants demonstrated elevated IL-6 (median 4.2 vs. 1.9 pg/mL; *p* = 0.002) and TNF-α (median 12.3 vs. 6.7 pg/mL; *p* = 0.01) in viraemic versus suppressed participants (Table 4). These findings align with evidence that persistent inflammation contributes to HIV-associated morbidity (10–12).

**Table 4:** Exploratory mechanistic findings (pilot subsets).

Parameter	Suppressed	Viraemic	*p*-value
Drug resistance (*n* = 50 viraemic)			
Any resistance mutation	—	43/50 (86%)	—
M184V/I (NRTI)	—	31/50 (62%)	—
K103N (NNRTI)	—	27/50 (54%)	—
Multi-class resistance	—	19/50 (38%)	—
Immune activation (*n* = 40)	*n* = 15	*n* = 25	
CD8+ CD38+ HLA-DR+ %, median (IQR)	12.4 (8.7–17.2)	28.6 (20.1–35.4)	<0.001
Correlation with CD4 (Spearman *ρ*)		−0.46	<0.01
Inflammatory biomarkers (*n* = 35)	*n* = 12	*n* = 23	
IL-6 (pg/mL), median (IQR)	1.9 (1.2–2.8)	4.2 (2.9–6.8)	0.002
TNF-α (pg/mL), median (IQR)	6.7 (4.2–9.1)	12.3 (8.4–18.6)	0.01

NRTI, nucleoside reverse transcriptase inhibitor; NNRTI, non-nucleoside reverse transcriptase inhibitor; IQR, interquartile range. Exploratory analyses in small pilot subsets; findings require validation.

### Factors associated with viral non-suppression

In multivariable analysis (*n* = 346), lower CD4 count was strongly associated with viral non-suppression (adjusted OR 0.53 per 100 cells/μL increase; 95% CI 0.43–0.66; *p* < 0.001). Age, sex, duration on ART, pregnancy status, and region were not significantly associated with non-suppression after adjustment (Table 5).

**Table 5:** Multivariable logistic regression for viral non-suppression (*n* = 346).

Variable	Adjusted OR	95% CI	*p*-value
CD4 count (per 100 cells/μL increase)	0.53	0.43–0.66	<0.001
Age (per 10 years)	1.12	0.89–1.41	0.34
Sex (male vs. female)	0.87	0.52–1.46	0.60
Duration on ART (per year)	1.03	0.96–1.11	0.42
Pregnancy (yes vs. no)	1.28	0.68–2.41	0.44
Region (ref: Southern)			
Central	1.34	0.71–2.53	0.37
Northern	1.52	0.79–2.92	0.21

Model includes site fixed effects; AIC = 389.2. OR, odds ratio; ART, antiretroviral therapy.

## Discussion

This multi-centre study reveals substantial gaps in HIV treatment outcomes at Nigerian tertiary care facilities, with viral suppression rates (26.9%) falling far below the UNAIDS 95% target and recent national programmatic estimates (6). The high prevalence of severe immunodeficiency (38.5%), discordant immune-virological responses (32.3%), and preliminary evidence of widespread drug resistance and immune activation suggest multiple interacting pathways to treatment failure in this population (25–29).

Our suppression rates contrast with recent Nigerian data showing national suppression approaching 95% (6). This discrepancy likely reflects the tertiary care setting, which concentrates patients with treatment challenges, suspected failure, and complex clinical presentations. Similar facility-level heterogeneity has been documented elsewhere in sub-Saharan Africa (20–22).

The finding that 25.7% of participants had viraemia despite preserved CD4 counts is clinically significant. This discordant pattern may represent early treatment failure before immunological decline, host factors conferring immune resilience, or measurement timing effects. Regardless of mechanism, these individuals remain at risk of disease progression and onward transmission, highlighting the importance of viral load monitoring over CD4-only approaches (20, 23).

The 86% prevalence of drug resistance mutations in our pilot subset, while requiring confirmation in larger studies, aligns with regional data showing high acquired resistance in failing patients (15–18). The predominance of M184V and K103N reflects selection by commonly used NRTI/NNRTI-based regimens and underscores the importance of resistance testing to guide regimen switches (16, 23, 24).

Elevated immune activation (CD38+HLA-DR+CD8+ T cells) and inflammatory biomarkers (IL-6, TNF-α) in viraemic participants are consistent with established pathophysiological mechanisms linking chronic immune activation to CD4 depletion and disease progression (9, 13, 14, 19). The inverse correlation between CD8 activation and CD4 count supports a potential mechanistic link, though causality cannot be established from cross-sectional data (30–35).

## Limitations

Several limitations warrant consideration. First, the cross-sectional design precludes causal inference and assessment of temporal relationships. Second, the tertiary care setting limits generalisability to primary care populations where treatment outcomes may differ. Third, mechanistic analyses were performed in small convenience subsets (*n* = 35–50), limiting statistical power and generalisability; these findings require validation in larger, systematically sampled populations. Fourth, adherence was not directly measured, precluding assessment of its contribution to treatment failure. Fifth, missing viral load (20.3%) and CD4 (25.1%) data may introduce selection bias. Sixth, single time-point sampling cannot distinguish persistent from transient viraemia. Despite these limitations, our findings provide important insights into treatment outcomes and potential failure mechanisms in a high-burden setting.

## Conclusion

HIV treatment outcomes at Nigerian tertiary care facilities fall substantially short of global targets, with high rates of viral non-suppression, severe immunodeficiency, and discordant immune-virological responses. Preliminary mechanistic data suggest contributions from drug resistance, immune activation, and inflammation. These findings highlight the need for enhanced viral load monitoring, resistance testing, and research into interventions targeting immune activation. Larger, longitudinal studies with systematic sampling are warranted to validate these mechanistic associations and inform targeted interventions to improve treatment outcomes in this high-burden setting.

## Data Availability

The original contributions presented in the study are included in the article/supplementary material, further inquiries can be directed to the corresponding author.

## References

[1] UNAIDS. The Path that Ends AIDS: 2023 UNAIDS Global AIDS Update. Geneva: Joint United Nations Programme on HIV/AIDS (2023).

[2] World Health Organization. HIV and AIDS: Fact Sheet. Geneva: WHO (2024).

[3] Mosha IH Nyondo GG Munishi CG Njiro BJ Bwire GM. Prevalence and factors associated with viral non-suppression in people living with HIV receiving antiretroviral therapy in sub-Saharan Africa: a systematic review and meta-analysis. Rev Med Virol. (2024) 34:e2540. doi: 10.1002/rmv.2540, 38708846 PMC11829566

[4] Stelzle D. Rangaraj A. Jarvis J. Razakasoa N.H. Low-Beer D. Doherty M. High prevalence of advanced HIV disease in sub-Saharan Africa: an analysis of household surveys. Conference on Retroviruses and Opportunistic Infections (CROI), Denver, (abstract 196), (2024).

[5] Onovo AA Adeyemi A Onime D Kalnoky M Kagniniwa B Dessie M . Estimation of HIV prevalence and burden in Nigeria: a Bayesian predictive modelling study. EClinicalMedicine. (2023) 62:102098. doi: 10.1016/j.eclinm.2023.102098, 37538543 PMC10393599

[6] Boyd MA Abutu A Bachanas P Chun HM Milligan K Dirlikov E. Reaching HIV epidemic control in Nigeria using a lower HIV viral load suppression cut-off. AIDS. (2023) 37:2081–5. doi: 10.1097/qad.0000000000003672, 37503650 PMC10990556

[7] Ford N Shubber Z Meintjes G Grinsztejn B Eholié SP Mills EJ. Advanced HIV as a neglected disease. N Engl J Med. (2024) 390:487–9. doi: 10.1056/NEJMp2311237 38314820

[8] Dzinamarira T Moyo E. Adolescents and young people in sub-Saharan Africa: overcoming challenges and seizing opportunities to achieve HIV epidemic control. Front Public Health. (2024) 12:1321068. doi: 10.3389/fpubh.2024.1321068, 38566795 PMC10985137

[9] Arrigoni FIF Spyer M Hunter P Boffito M Sabin CA Post FA. Immune activation biomarkers in people living with HIV after second-line ART failure in sub-Saharan Africa. AIDS. (2023) 37:913–23. doi: 10.1097/QAD.000000000000345236723505

[10] Castillo-Mancilla JR Brown TT Erlandson KM Palella FJ Jr Gardner EM Macatangay BJ . Suboptimal adherence to combination antiretroviral therapy is associated with higher levels of inflammation despite HIV suppression. Clin Infect Dis. (2023) 63:1661–1667. doi: 10.1093/cid/ciw650 PMC5146724 27660234

[11] Tenorio AR Zheng Y Bosch RJ Krishnan S Rodriguez B Hunt PW . Soluble markers of inflammation and coagulation but not T-cell activation predict non-AIDS-defining morbid events during suppressive antiretroviral treatment. J Infect Dis. (2023) 227:381–9. doi: 10.1093/infdis/jiu254 24795473 PMC4192039

[12] Sandler NG Wand H Roque A Law M Nason MC Nixon DE . Plasma levels of soluble CD14 independently predict mortality in HIV infection. J Infect Dis. (2021) 210:1248–1257. doi: 10.1093/infdis/jiu254 PMC3071127 21252259

[13] Hunt PW Lee SA Siedner MJ. Immunologic biomarkers, morbidity, and mortality in treated HIV infection. J Infect Dis. (2021) 214:544–550. doi: 10.1093/infdis/jiw275 PMC5021241 27625430

[14] Deeks SG Tracy R Douek DC. Systemic effects of inflammation on health during chronic HIV infection. Immunity. (2023) 39:633–45. doi: 10.1016/j.immuni.2013.10.001 PMC4012895 24138880

[15] Kavuraya AN Mwita S Lyimo E Mboya IB Mrema J Kalinga A. The genetic diversity and drug resistance patterns of HIV-1 *pol* gene in East Africa. Adv Virol. (2025) 2025:102.43. 10.1155/av/2801818 PMC12539661 41140464

[16] Wensing AM Calvez V Ceccherini-Silberstein F Charpentier C Günthard HF Paredes R . 2022 update of the drug resistance mutations in HIV-1. Top Antivir Med. (2022) 30:559–74. 36375130 PMC9681141

[17] Hauser A Goldstein F Reichmuth ML Kouyos RD Wandeler G Egger M . Acquired HIV drug resistance mutations on first-line antiretroviral therapy in southern Africa: systematic review and Bayesian evidence synthesis. J Infect Dis. (2022) 148:72–82. doi: 10.1016/j.jclinepi.2022.04.021 PMC9388696 35192922

[18] Obasa AE Ambikan AT Gupta S Neogi U Jacobs GB. Increased acquired protease inhibitor drug resistance mutations in minor HIV-1 quasispecies from infected patients suspected of failing on national second-line therapy in South Africa. BMC Infect Dis. (2021) 21:214. doi: 10.1186/s12879-021-05969-x 33632139 PMC7908688

[19] Rueda CM Velilla PA Chougnet CA Montoya CJ Rugeles MT. Incomplete normalization of CD8+ T cell function in virologically suppressed HIV-infected adults receiving antiretroviral therapy. AIDS Res Ther. (2022) 19:52. doi: 10.1186/s12981-022-00450-536384677

[20] Tsega TD Tsega SS Gebeyehu AA Yemata GA Anteneh RM Yeshiwas AG . Viral load suppression rate of third-line antiretroviral therapy and its association with gender among HIV patients after second-line treatment failure in Africa: a systematic review and meta-analysis. BMC Infect Dis. (2025) 25:158. doi: 10.1186/s12879-025-10576-4, 39901068 PMC11792664

[21] Kyere GA Vechey GA Charles-Unadike VO Tarkang EE. Trends in viral load suppression among HIV patients on antiretroviral therapy (ART) at Asante Mampong municipal hospital, Ghana: 2019–2023. BMC Infect Dis. (2024) 24:1170. doi: 10.1186/s12879-024-10072-1, 39415134 PMC11483983

[22] Olagunju FA Oninla SO Adeyemo SC Fadahunsi KP Odeyemi AO Olabode ED. Predictors of virological outcomes in patients with HIV on antiretroviral therapy in Osogbo, Nigeria: a cross-sectional study. BMC Infect Dis. (2025) 25:402. doi: 10.1186/s12879-025-11084-1 40413409 PMC12102910

[23] Siedner MJ Moosa MYS McCluskey S Gilbert RF Pillay S Aturinda I . Resistance testing for management of HIV virologic failure in sub-Saharan Africa: an unblinded randomized controlled trial. Ann Intern Med. (2021) 174:1683–92. doi: 10.7326/M21-2229, 34698502 PMC8688215

[24] Gandhi RT Bedimo R Hoy JF Landovitz RJ Smith DM Eaton EF . Antiretroviral drugs for treatment and prevention of HIV infection in adults: 2022 recommendations of the international antiviral society-USA panel. JAMA. (2023) 329:63–84. doi: 10.1001/jama.2022.22246, 36454551

[25] Mitchell BI Yazel Eiser IE Kallianpur KJ Gangcuangco LM Chow DC Ndhlovu LC . Dynamics of peripheral T cell exhaustion and monocyte subpopulations in neurocognitive impairment and brain atrophy in chronic HIV infection. J Neurovirol. (2024) 30:489–99. doi: 10.1007/s13365-024-01223-w, 38949728 PMC11846764

[26] Haile GS Berhe AA Gebremedhin GB. Burden and associated factors of virological failure among people living with HIV in sub-Saharan Africa: a systematic review and meta-analysis. PLoS One. (2022) 17:e0265599. doi: 10.1371/journal.pone.0265599 35416596

[27] Rhee SY Kassaye SG Engelbrecht S Jordan MR Shafer RW. Global surveillance of HIV-1 drug resistance and prevalence of pre-treatment drug resistance. Lancet Microbe. (2022) 3:e554–64. doi: 10.1016/S2666-5247(22)00103-6 35544100 PMC9095989

[28] National Agency for the Control of AIDS (NACA). Nigeria HIV/AIDS Indicator and Impact Survey (NAIIS) 2018: Technical Report. Abuja: Federal Ministry of Health (2019).

[29] Rugemalila J Kamori D Kunambi P Mizinduko M Sabasaba A Masoud S . HIV virologic response, patterns of drug resistance mutations and correlates among adolescents and young adults: a cross-sectional study in Tanzania. J Pediatr Infect Dis Soc. (2023) 18:e0281528. doi: 10.1371/journal.pone.0281528 PMC9949668 36821538

[30] Etta EM Mavhandu L Manhaeve C McGonigle K Jackson P Rekosh D . High level of HIV-1 drug resistance mutations in patients with unsuppressed viral loads in rural northern South Africa. AIDS Res Ther. (2017) 14:36. doi: 10.1186/s12981-017-0161-z 28750647 PMC5531022

[31] Sigaloff KC Hamers RL Wallis CL Kityo C Siwale M Ive P . Unnecessary antiretroviral treatment switches and accumulation of HIV resistance mutations; two arguments for viral load monitoring in Africa. J Acquir Immune Defic Syndr. (2021) 58:23–31. doi: 10.1097/QAI.0b013e318227fc34 21694603

[32] Masenga SK Mweene BC Luwaya E Muchaili L Chona M Kirabo A. HIV-host cell interactions. Cells. (2023) 12:1351. doi: 10.3390/cells12101351, 37408185 PMC10216808

[33] Hurbans N Naidoo P. Efficacy, safety, and tolerability of dolutegravir-based ART regimen in Durban, South Africa: a cohort study. BMC Infect Dis. (2024) 24:343. doi: 10.1186/s12879-024-09202-6, 38515041 PMC10958909

[34] Chakravarty J Srivastva S Kushwaha AK Pal A. Long-term treatment outcome and mutational analysis of patients on third-line antiretroviral therapy in programmatic conditions. J Antimicrob Chemother. (2023) 78:1480–7. doi: 10.1093/jac/dkad111, 37042390

[35] Bwire GM Kamori D Mpondo BCT Mmbaga BT Chiduo MG Mrema M . High viral suppression and detection of dolutegravir-resistance associated mutations in treatment-experienced Tanzanian adults living with HIV-1 in Dar Es Salaam. J Antimicrob Chemother. (2023) 78:779–87. doi: 10.1093/jac/dkad381 37993493 PMC10665358

